# Epidemiological Characteristics of Varicella in Anhui Province, China, 2012-2021: Surveillance Study

**DOI:** 10.2196/50673

**Published:** 2024-04-05

**Authors:** Kun Xuan, Ning Zhang, Tao Li, Xingya Pang, Qingru Li, Tianming Zhao, Binbing Wang, Zhenqiu Zha, Jihai Tang

**Affiliations:** 1 Anhui Provincial Center for Disease Control and Prevention Hefei, Anhui Province China; 2 School of Health Management Anhui Medical University Hefei China

**Keywords:** varicella, incidence, epidemiology, spatial autocorrelation, contagious disease, chicken pox, varicella zoster virus, China

## Abstract

**Background:**

Varicella is a mild, self-limited disease caused by varicella-zoster virus (VZV) infection. Recently, the disease burden of varicella has been gradually increasing in China; however, the epidemiological characteristics of varicella have not been reported for Anhui Province.

**Objective:**

The aim of this study was to analyze the epidemiology of varicella in Anhui from 2012 to 2021, which can provide a basis for the future study and formulation of varicella prevention and control policies in the province.

**Methods:**

Surveillance data were used to characterize the epidemiology of varicella in Anhui from 2012 to 2021 in terms of population, time, and space. Spatial autocorrelation of varicella was explored using the Moran index (Moran *I*). The Kulldorff space-time scan statistic was used to analyze the spatiotemporal aggregation of varicella.

**Results:**

A total of 276,115 cases of varicella were reported from 2012 to 2021 in Anhui, with an average annual incidence of 44.8 per 100,000, and the highest incidence was 81.2 per 100,000 in 2019. The male-to-female ratio of cases was approximately 1.26, which has been gradually decreasing in recent years. The population aged 5-14 years comprised the high-incidence group, although the incidence in the population 30 years and older has gradually increased. Students accounted for the majority of cases, and the proportion of cases in both home-reared children (aged 0-7 years who are not sent to nurseries, daycare centers, or school) and kindergarten children (aged 3-6 years) has changed slightly in recent years. There were two peaks of varicella incidence annually, except for 2020, and the incidence was typically higher in the winter peak than in summer. The incidence of varicella in southern Anhui was higher than that in northern Anhui. The average annual incidence at the county level ranged from 6.61 to 152.14 per 100,000, and the varicella epidemics in 2018-2021 were relatively severe. The spatial and temporal distribution of varicella in Anhui was not random, with a positive spatial autocorrelation found at the county level (Moran *I*=0.412). There were 11 districts or counties with high-high clusters, mainly distributed in the south of Anhui, and 3 districts or counties with high-low or low-high clusters. Space-time scan analysis identified five possible clusters of areas, and the most likely cluster was distributed in the southeastern region of Anhui.

**Conclusions:**

This study comprehensively describes the epidemiology and changing trend of varicella in Anhui from 2012 to 2021. In the future, preventive and control measures should be strengthened for the key populations and regions of varicella.

## Introduction

Varicella, also known as chicken pox, is a highly contagious, self-limited disease caused by varicella-zoster virus (VZV) that commonly infects children and could become reactivated following primary infection to induce herpes zoster (HZ), or shingles, infection in the future [1]. As a human-specific pathogenic alphaherpesvirus, primary VZV infection first acts on the upper respiratory mucosa with an incubation period of 10-21 days, followed by a generalized vesicular rash, which is accompanied by fever and headache [2,3]. VZV will remain latent in the human ganglia after the primary infection, which may then become reactivated to induce HZ, a localized, painful vesicular rash that can lead to the development of postherpetic neuralgia in some individuals [4,5]. Although varicella usually presents with mild symptoms, the World Health Organization (WHO) conservatively estimated that 4.2 million hospitalizations were associated with severe varicella complications annually worldwide [6], and that use of the vaccine has reduced the disease burden of varicella to a certain extent.

During the 1960s and 1970s, Japanese scientists developed a live attenuated varicella vaccine with the Oka VZV strain after several cell cultures and passages, which was subsequently used for the active immunization of varicella in many countries [7,8]. Varicella vaccines are commonly used globally in monovalent form or combined with the measles, mumps, and rubella vaccine; varicella vaccines have an overall effectiveness of 81% and effectiveness against moderate or severe varicella of 98% [9]. The WHO recommended that varicella vaccination coverage should be maintained at more than 80% and considered including varicella vaccination in the routine childhood immunization program [6]. Currently, 50 WHO member countries or territories have introduced the varicella vaccine in the routine vaccination schedule [10]. In countries such as the United States, Germany, and Costa Rica, the incidence and hospitalization rates of varicella have significantly reduced compared to those of the prevaccination era [11-14]; thus, the varicella vaccination program has had a positive impact on the epidemiology and disease burden of varicella. However, varicella vaccination is voluntary and self-paying in most areas of China, with few areas providing the vaccine for free and the two-dose schedule has not yet been introduced nationwide. The varicella vaccination rate in China was estimated to be 61% [15], and the reported incidence of varicella increased from 55 per 100,000 people in 2016 to 70 per 100,000 people in 2019, with an average of 1000 outbreaks occurring annually [16]. In addition, reports from some regions of China have shown that the incidence of varicella has increased by approximately 40% annually [17-19]. However, related data have not yet been reported for Anhui Province.

Spatiotemporal investigations based on geographic information systems have been increasingly used in modern epidemiology research [20], which could help to visualize the incidence trends and spatial distributions of infectious diseases, providing a new framework for analyzing their spatiotemporal changes and transmission patterns. Previous studies demonstrated the spatiotemporal aggregation and spatial correlation patterns of varicella occurrence [19,21]. However, no study has yet focused on the epidemiological characteristics or spatial and temporal distributions of varicella in Anhui. Since Anhui is an inland province in the east of China with vast differences in population distribution and geographical characteristics, a systematic epidemiological analysis of the current varicella prevalence could provide a basis for future studies and strategies targeting varicella in Anhui.

Toward this end, the aim of this study was to analyze the epidemiological characteristics of varicella in Anhui using the data of varicella incidence from 2012 to 2021, combined with spatiotemporal epidemiological methods. These findings can provide a basis for the formulation of varicella prevention and control policies and immunization strategies.

## Methods

### Setting

Anhui Province covers a total area of 140,100 km^2^, located at longitude 114°54'-119°37' E and latitude 29°41'-34°38' N. Anhui covers the jurisdiction of 16 cities and 104 county-level administrative regions and is part of the Yangtze River Delta region of China. Anhui lies in the transition zone between the warm temperate zone and subtropics, with an average annual temperature of 14-17 °C. The Yangtze River and the Huai River run through the province, dividing the province into three parts, with an overall high elevation in the southwest and low elevation in the northeast. The population of Anhui was estimated at approximately 61.13 million by the end of 2021, showing a greater density in the north than in the south of the province.

### Data Sources

Data on reported varicella cases and population statistics were obtained from the China Information System for Disease Control and Prevention (CISDCP), which is the world’s largest internet-based disease reporting system that was established by the Chinese government after the 2003 SARS epidemic. The CISDCP is based on the real-time reporting of individual cases and the relevant information of each case can be obtained, such as age, sex, occupation, and the specific location of the onset [22]. Clinical- and laboratory-confirmed cases of varicella reported in the CISDCP were selected for analysis in this study.

### Descriptive Analysis

The annual and monthly incidence rates of varicella from 2012 to 2021 were collected and epidemic curves were plotted to reveal the incidence peaks. The distribution characteristics of varicella according to variations in age, sex, and occupation were also analyzed. The incidence of varicella in each county or city was associated with geographical information according to the administrative district code, and annual incidence maps were plotted to describe the distribution of varicella in Anhui. All statistical analyses and data visualization were performed using R Studio software (version 4.1.1).

### Spatial Autocorrelation Analysis

GeoDa (version 1.20) was used to perform the spatial autocorrelation analysis. The Moran index (Moran *I*) reflects the correlation of attribute values in adjacent areas and can be used to judge the overall spatial distribution [23]. The Moran *I* value ranges from –1 to 1, indicating a stronger negative or positive spatial autocorrelation, respectively. In this study, the spatial weight matrix was constructed according to the first-order Queen adjacency rule and the significance test of spatial autocorrelation was carried out. The null hypothesis was that varicella is randomly distributed in Anhui. The hypothesis test of local indicators of spatial association (LISA) [24] was performed and the Moran scatter plot was used to explore the association pattern of hotspot areas. The local association pattern can be divided into four categories: high-high, high-low, low-high, and low-low. The LISA significance test was performed using 999 permutations at a probability level of .05.

### Space-Time Scan Analysis

The space-time scan statistic is defined by a cylindrical window with a circular (or elliptic or network-based) geographic base and a height corresponding to time. The base is defined as a circular window with changing radius size centered on several possible grid points throughout the study area, while the height reflects the time period of potential clusters. The cylindrical window then moves in space and time so that every possible geographic location and size for each possible time period is accessed. In this process, an infinite number of overlapping cylinders of different sizes and shapes could be obtained, jointly covering the entire study area with each cylinder reflecting a possible cluster. For this study, we adopted the discrete Poisson model described by Kulldorff [25] to conduct a retrospective space-time scan analysis of the spatiotemporal aggregation of varicella. Maximum-likelihood estimation was performed for the locations and sizes of all cylindrical windows, and the alternative hypothesis was that the incidences were higher in the windows than in the outside regions. The *P* value of the likelihood ratio test was obtained by 999 Monte Carlo simulations based on a probability level of .05. The cylindrical window with the maximum-likelihood ratio was considered to be the most likely cluster and the secondary cluster was sorted according to the size of likelihood ratio test statistics. The maximum spatial cluster size and temporal cluster size were set to 20% of the population at risk and 30% of the study period, respectively. The space-time scan analysis was performed using SaTScan (version 10.1).

### Ethical Considerations

This study used monitoring data from the CISDCP and no sensitive information was retrieved about the cases or patients. Since this study did not involve any human subjects, it did not require notification to the Ethics Committee according to national ethical review regulations [26].

## Results

### General Epidemiological Characteristics

**Figure 1:**
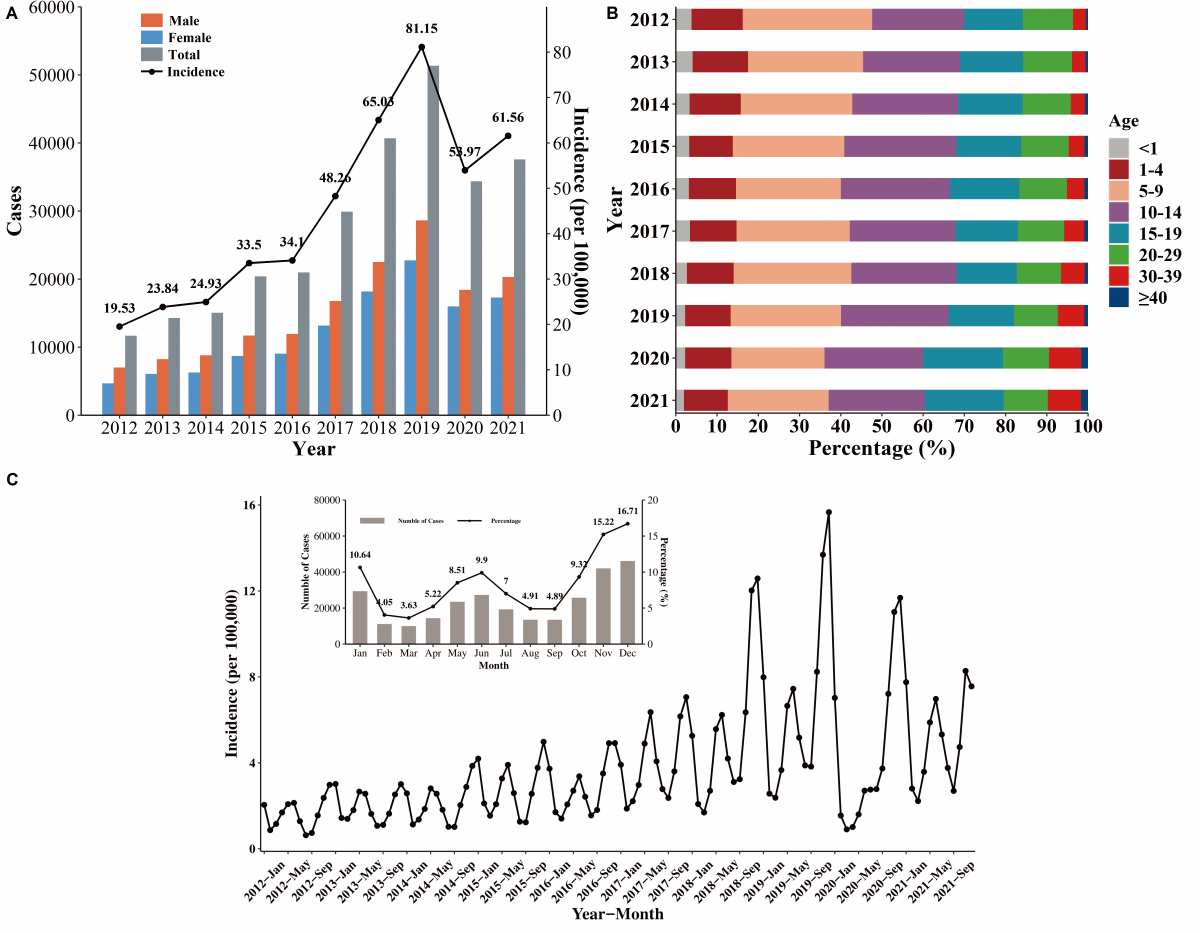
(A) Incidence of varicella and number of cases by sex, 2012-2021. (B) Age (years) distribution of varicella, 2012-2021. (C) Monthly incidence of varicella in Anhui Province, 2012-2021.

From 2012 to 2021, there were 276,115 cases of varicella and 4 deaths in Anhui, and the average annual incidence was 44.8 per 100,000. Overall, there was an increasing trend of varicella incidence between 2012 and 2019, followed by a decline in 2020 and a slight rebound in 2021. The proportion of male cases among the total number of new annual cases showed a decreasing trend over the decade, whereas the number of female cases gradually increased (*χ*^2^_1_=269.18, *P*_for trend_ <.001); the overall sex ratio of cases was approximately 1.26. Details are shown in Table 1 and Figure 1A.

The main age of varicella incidence was 5-14 years, accounting for more than 50% of all cases. The proportion of varicella cases decreased gradually in people aged 0-9 years, whereas there was an increasing trend among those 30 years or older (Figure 1B). In addition, students (ie, children and adolescents 6 years and older who are already enrolled in school) accounted for approximately 60% of reported cases, and the proportion of cases in home-reared children and kindergarten children (aged 3-6 years) increased by 5% and decreased by 9%, respectively (Table 1).

The epidemic curve showed the typical seasonal distribution of varicella with peak incidence occurring twice annually. The incidence was usually higher in the winter peak (November to January of the following year) than in the summer peak (May to June). Only the winter peak occurred in 2020 (Figure 1C).

**Figure 2:**
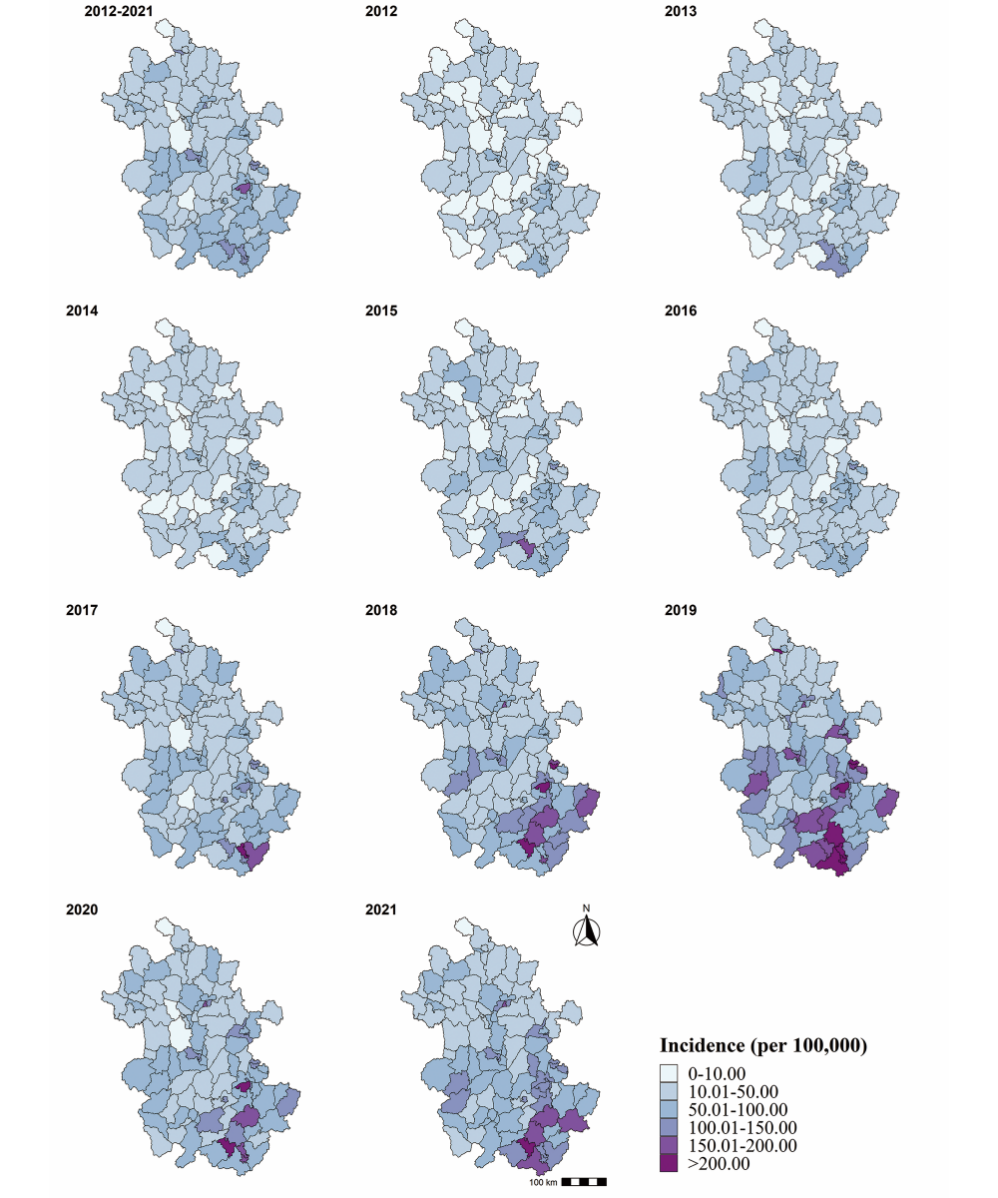
Annual incidence maps of varicella in Anhui Province, 2012-2021.

In terms of the geographical distribution, the incidence of varicella was commonly higher in southern Anhui than in northern Anhui from 2012 to 2021. The high-prevalence areas were the cities of Huangshan, Wuhu, and Ma’anshan, and the lower-prevalence areas were the cities of Huainan, Suzhou, and Anqing (see Multimedia Appendix 1). The incidence in most areas was lower than 50 per 100,000 from 2012 to 2016, whereas between 2018 and 2021, there were some relatively severe varicella epidemics along with clear regional differences (Figure 2). The average incidence at the county level ranged from 6.61 to 152.14 per 100,000.

**Table 1:** Characteristics of the varicella population distribution in Anhui Province from 2012 to 2021.

Variables		2012-2013 (n=25,934), n (%)	2014-2015 (n=35,410), n (%)	2016-2017 (n=50,848), n (%)	2018-2019 (n=91,994), n (%)	2020-2021 (n=71,929), n (%)
**Sex**						
	Male	15,224 (58.7)	20,464 (57.8)	28,672 (56.4)	51,105 (55.6)	38,675 (53.8)
	Female	10,710 (41.3)	14,946 (42.2)	22,176 (43.6)	40,889 (44.4)	33,254 (46.2)
	Male/female	1.42	1.37	1.29	1.25	1.16
**Age (years)**						
	0-1	1036 (4.0)	1177 (3.3)	1693 (3.3)	2290 (2.5)	1520 (2.1)
	1-4	3366 (13.0)	4012 (11.3)	5771 (11.3)	10,261 (11.2)	7871 (10.9)
	5-9	7641 (29.5)	9577 (27.0)	13,555 (26.7)	25,357 (27.6)	16,951 (23.6)
	10-14	5952 (23.0)	9415 (26.6)	13,225 (26.0)	23,805 (25.9)	16,975 (23.6)
	15-19	3850 (14.8)	5545 (15.7)	8059 (15.8)	14,060 (15.3)	13,857 (19.3)
	20-29	3114 (12.0)	4111 (11.6)	5757 (11.3)	9811 (10.7)	7856 (10.9)
	≥30	975 (3.7)	1573 (4.5)	2788 (5.6)	6410 (6.9)	6899 (9.6)
**Classification**						
	Home-reared children^a^	2397 (9.2)	3367 (9.5)	5035 (9.9)	11,653 (12.7)	9583 (13.3)
	Nursery/kindergarten children (3-6 years old)	4394 (16.9)	5240 (14.8)	6579 (12.9)	8312 (9.0)	5678 (7.9)
	Student	15,249 (58.8)	21,458 (60.6)	30,849 (60.7)	56,425 (61.3)	43327 (60.2)
	Farmer	1107 (4.3)	1654 (4.7)	2799 (5.5)	4494 (4.9)	3447 (4.8)
	Others	2787 (10.8)	3691 (10.4)	5586 (11.0)	7110 (12.1)	9894 (13.8)

^a^Home-reared children refer to infants and young children (from birth to 7 years old) who are not sent to nursery or kindergarten and are raised and educated at home.

### Spatial Autocorrelation

**Figure 3:**
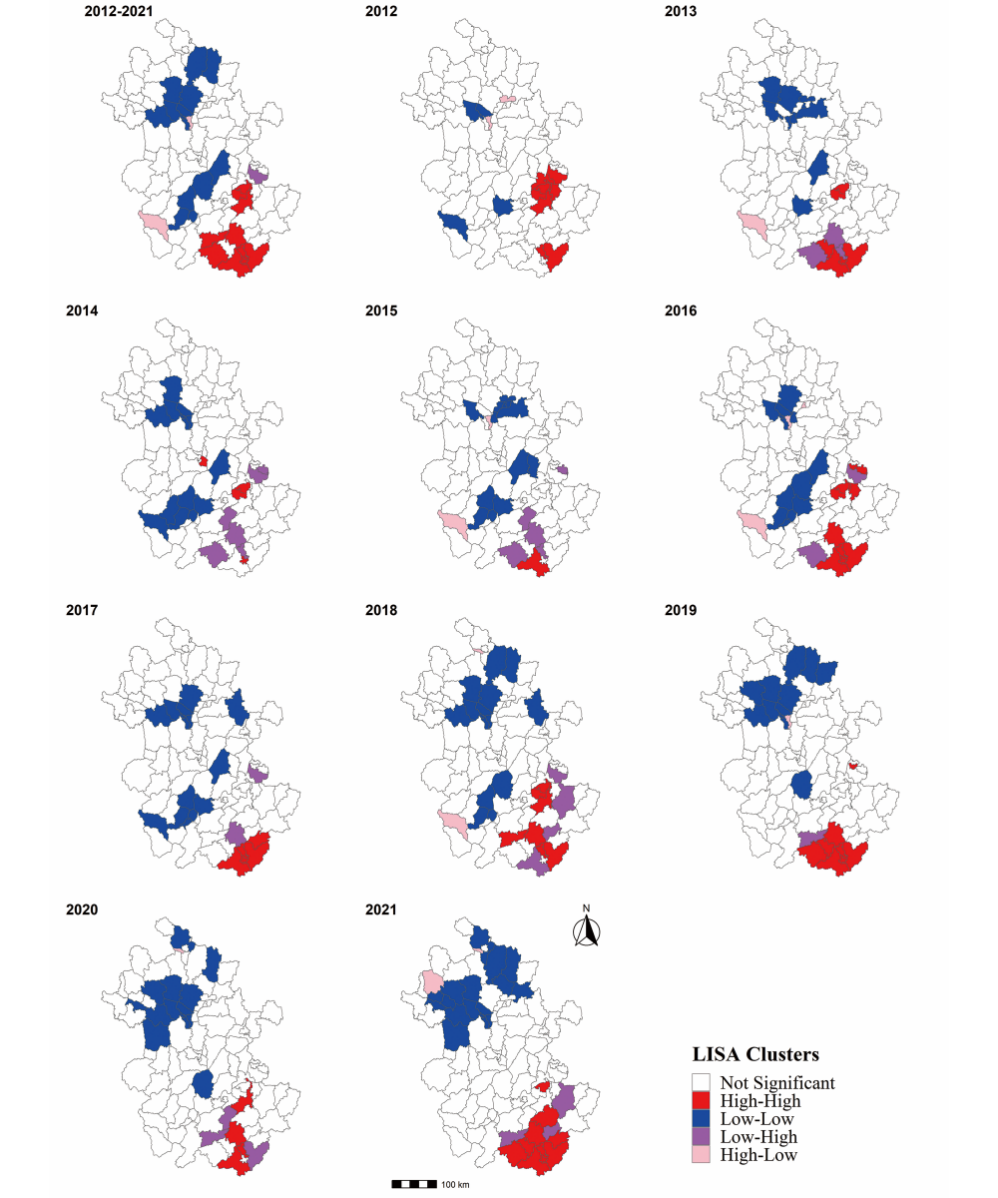
Spatial autocorrelation local indicators of spatial association (LISA) cluster maps of varicella in Anhui Province, 2021-2021.

Globally, a positive spatial autocorrelation of varicella incidence was found at the county level from 2012 to 2021, with Moran *I* of 0.412; this positive correlation was significant throughout the observation period, with Moran *I* ranging from 0.186 to 0.451 (Table 2). All of the hotspot clusters (high-high) were distributed in the southern region, including six districts and five counties in Huangshan, Wuhu, and Chizhou. The low-low clusters were mainly identified in Huainan, Anqing, and Suzhou. The high-low and low-high clusters included Tianjia’an District, Taihu, and Dangtu County (Figure 3).

**Table 2:** Global spatial autocorrelation analysis of varicella in Anhui Province, 2012-2021.

Year	Moran index	Mean (SD)	*Z* value	*P* value
2012	0.313	–0.0109 (0.0644)	5.029	.001
2013	0.253	–0.0095 (0.0621)	4.226	.001
2014	0.186	–0.0086 (0.0649)	2.993	.003
2015	0.204	–0.0121 (0.062)	3.485	.003
2016	0.347	–0.0107 (0.0622)	5.762	.001
2017	0.319	–0.0098 (0.0625)	5.264	.001
2018	0.282	–0.0097 (0.0593)	4.913	.001
2019	0.409	–0.0118 (0.0612)	6.877	.001
2020	0.282	–0.0084 (0.0621)	4.681	.001
2021	0.451	–0.0106 (0.0622)	7.424	.001
Overall	0.412	–0.0132 (0.0626)	6.795	.001

### Spatiotemporal Clusters

**Figure 4:**
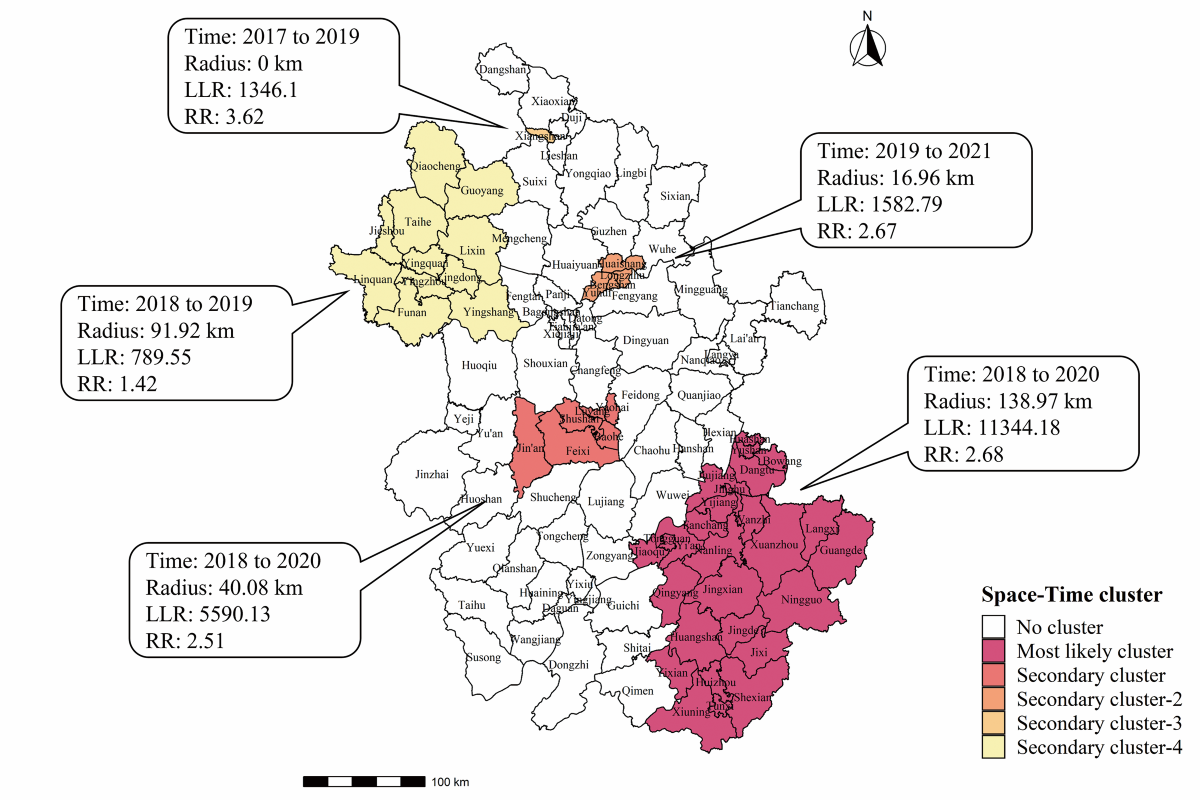
Spatiotemporal clustering maps of varicella in Anhui province, 2012-2021. LLR: likelihood ratio; RR: relative risk.

Space-time scan analysis identified five possible clusters of areas where varicella incidence was higher than that in other areas in the specific time period. The most likely cluster was located in southeastern Anhui, including parts of Ma’anshan, Wuhu, Huangshan, Tongling, and Chizhou and the whole territory of Xuancheng. The four secondary clusters were mainly distributed in Hefei, Bengbu, Fuyang, and the Xiangshan district of Huaibei (Figure 4).

## Discussion

### Principal Findings

In this study, we comprehensively described the epidemiology of varicella in Anhui over the last decade. To our knowledge, this is the first study to analyze the changing epidemiological characteristics and identify the incidence hotspot areas of varicella from a province perspective. The reported incidence of varicella in Anhui increased by approximately 20% annually from 2012 to 2019, with a 10-year average incidence of 44.8 per 100,000, which is largely consistent with the rising trend in other regions of China [16,27,28]. The incidence peaked in 2019 at 81.2 per 100,000 people, which is higher than the national average but is lower than the averages for Guizhou, Hangzhou, and Dalian for the same period [28-30]. The higher incidence of varicella in Anhui may be related to the increased sensitivity of infectious disease surveillance in recent years but also may be related to the insufficient coverage of vaccination. In the United States, the routine varicella vaccination program began in 1995, and the Advisory Committee on Immunization Practices recommended adding a second dose for children 4-6 years of age in 2007 [31]; there has been a 97% reduction in varicella incidence in 2019 compared to that prior to the implementation of routine vaccination [32]. Japan introduced routine vaccination with two doses of varicella vaccine in 2014, and the number of varicella hospitalizations decreased by 77% in 2017, including an 88% decrease in hospitalization of children aged 1-4 years [33]. By 2019, the incidence of varicella in Japan had decreased by 78% from that reported in 2000-2011 [34]. In South Korea and Turkey, in which one-dose routine immunization was implemented, the incidence, hospitalization, and complication rates of varicella were significantly reduced due to high vaccination rates [14,35]. The varicella vaccine is not currently included in the routine immunization program in China and there is no universal two-dose varicella vaccination, which may explain the high disease burden of varicella in Anhui. 

Similar to previous studies, there were more cases of varicella in males than females [16,19]. However, the sex difference has narrowed gradually in recent years, which was also found in the study in Chongqing, China [19]. This may be caused by the widespread transmission of VZV in susceptible populations as the community has not yet established immune barriers. The proportion of varicella cases among children and adolescents under 14 years of age decreased slightly, whereas the proportion among adults 30 years and older tended to increase in Anhui. More children are now vaccinated compared to the historical proportion, and the vaccine is demonstrated to be effective in reducing the risk of infection in younger age groups. This study found a shift in the age of onset to older age groups, which is consistent with previous studies [36,37]. However, this outcome differs from other studies that did not observe an increase in the average age of varicella incidence, likely due to herd immunity obtained from high vaccine coverage [14,38,39]. Nevertheless, enhancing surveillance of changes in varicella prevalence among adults in Anhui, especially those over 30 years of age, is warranted in the future. In addition, the proportion of varicella cases among kindergarten children gradually decreased, whereas the proportion increased among home-reared children, which slightly differs from the results of a previous study in Chongqing [19]. More children were vaccinated before entering kindergarten according to confirmation of vaccination certificates, which also provides considerable protection despite the risk of clustered outbreaks. Suspected cases of varicella can also be detected in a timely manner through routine morning and noon physical examinations, thereby reducing the impact on other children. In general, home-reared children are less likely to be vaccinated because there is not a preadmission vaccination certificate requirement as is the case for enrolling in kindergarten. Moreover, since home-reared children typically accompany their guardians in their living and socializing activities, they are more likely to contact a wider variety of people compared to kindergarten children and do not have the immunity from vaccination; thus, home-reared children are more likely to come into contact with VZV-infected people, making them more susceptible to VZV infection. This suggests that more attention should be paid to home-reared children to improve their vaccination rate and protection, along with further education of their guardians regarding varicella in parallel with strengthening school outbreak surveillance.

Varicella showed a seasonal rise in winter and summer, which was consistent with previous studies [19,21]. In 2020, the incidence of varicella significantly declined and only a predominate winter peak occurred; this phenomenon has also been observed for other respiratory infectious diseases. The incidence of notifiable respiratory infectious diseases in China decreased significantly in 2020, with a 65% decrease in both measles and influenza [40]. Following the COVID-19 pandemic, many public health measures, including wider lockdowns, wearing masks, and social distancing, were taken to reduce the transmission of SARS-CoV-2 via droplets and aerosols [41]. These measures may have reduced the exposure to other respiratory viruses. In addition, concerns about COVID-19 infection and increased awareness of self-protection have led to fewer reports from health care facilities. According to the seasonal pattern of varicella (not considering 2020), health education, vaccination, and the sensitivity of surveillance should be strengthened during the low-incidence period to help reduce outbreaks and more severe prevalence during the high-incidence season.

The spatial and temporal distributions of varicella were not random in Anhui. Although spatiotemporal clusters of varicella have occurred in the cities of Bengbu, Huaibei, and Fuyang in the northern region of Anhui from 2017 to 2021, on the whole, the reported incidence in southern Anhui was higher than that in northern Anhui. Several studies found that respiratory infectious diseases showed patterns of high-risk clusters in remote rural or urban-rural transition zones where economic, educational, and medical resources are often lacking [21,42,43]. However, the most likely clusters of varicella in Anhui were located in the more economically developed areas of Hefei and in the southeast region of the province. Geographically, Anhui is connected to Jiangsu Province in the east and Zhejiang Province in the southeast, and the prevalence of varicella was higher in these two adjacent provinces than in Anhui, whereas the incidence was lower in the Henan and Shandong provinces north of Anhui [16,44]. There has been more frequent movement of the population in Anhui, mainly to Jiangsu and Zhejiang, in the past few years than in other provinces [45]. This may be one of the reasons for the inconsistency between the characteristics of high-risk clusters in this study and in previous studies. For example, Huangshan and Wuhu, which are located in the southeast of Anhui, were not only the high-incidence areas of varicella but were also high-high clusters, and the most likely spatiotemporal clusters were also distributed in these cities and their surroundings. Alternatively, there may be differences in surveillance sensitivity, vaccination rates, and geographic and climatic characteristics in different areas of Anhui, resulting in the observed variable spatial distribution pattern of varicella. By contrast, northern Anhui is located in the southern part of the North China Plain with a relatively higher population density and mobility than other areas of the province, which would theoretically facilitate the transmission of respiratory infectious diseases. Considering the high underestimation rate of varicella reporting in China [44], the actual disease burden in these apparently low-incidence areas may actually be far greater than indicated in our analysis. Therefore, the true reasons for the overall difference in incidence between northern and southern Anhui need to be thoroughly explored. 

Moreover, the high-low and low-high clusters identified by spatial autocorrelation analysis are perhaps of greater concern than clusters of hot or cold spots. In other words, more attention needs to be paid to the surrounding varicella incidence to avoid the inflow of the epidemic from the high-incidence areas (Dangtu County) or the increased incidence in the surrounding areas due to the high local incidence (Tianjia’an District and Taihu County), providing evidence for the future focus of varicella prevention and control strategies.

Although vaccination can have a positive impact on the epidemiology of varicella, some studies found that universal routine immunization with varicella vaccine increased the incidence of HZ [46,47]. Although caused by the same virus, these two diseases were not analyzed together in this study due to the lack of data on HZ. Humoral immunity persists in individuals with prior varicella infection; however, cell-mediated immunity, which plays an important role in the development of HZ, typically wanes with age [47,48]. Exposure to wild-type varicella cases could enhance cell-mediated immunity against VZV and maintain the immune effect for a longer time, whereas the opportunity for such exogenous enhancement decreases when varicella vaccination rates increase, which may account for the increased incidence of HZ [49]. Varicella is closely related to the epidemiology of HZ; thus, attention should be paid to enhancing the surveillance of HZ. Although the vaccine reduces the disease burden of varicella, the disease burden resulting from the increased incidence of HZ may offset some of the economic benefits of varicella vaccination, which should be taken into account when including varicella into routine immunization programs. According to the varicella vaccine immunization strategy in Anhui, since June 2021, the first dose of varicella vaccine is recommended at the age of 1 year and the second dose is recommended at the age of 4 years. Therefore, the single- and double-dose vaccination rates and the effects of administering two doses of the vaccine also warrant further study.

### Limitations

There are some limitations of this study. First, although substantial disease information has been provided through the CISDCP surveillance, there are still some shortcomings in the surveillance of varicella in Anhui Province. The proportion of cases underreported after mandatory reporting is likely to be small [50,51] and is unlikely to affect the epidemiology of the disease. Some regions in China already require the reporting of varicella cases within 24 hours [19,29], although this is not mandatory in Anhui and the actual data on underreporting are currently unavailable, which may have led to an underestimation of the incidence and possible alteration of the epidemiological characteristics of varicella. Therefore, more comprehensive varicella surveillance data are needed to assess the effectiveness of vaccination and to guide future varicella control and immunization strategies. Second, scan statistics based on circular windows often produce clusters that are larger than they actually are and can fail to identify irregular clusters; therefore, varicella clusters identified using flexible scan statistics may be more practical [52]. Finally, information on factors affecting the spread of infectious diseases, such as economy, population density, vaccination rates, and climate, was not considered, as this work only described the aggregation phenomenon of varicella; thus, the potential underlying causes need to be further studied.

### Conclusion

This study provides detailed epidemiological characteristics and changes of varicella from the perspectives of population, time, and space based on the reported incidence data in Anhui from 2012 to 2021. Measures should be taken in the future to strengthen varicella prevention and control in the key populations and regions identified in this study.

## Appendix

Multimedia Appendix 1 Total incidence of varicella in each city of Anhui Province, 2012-2021.

## Abbreviations

CISDCP: China Information System for Disease Control and Prevention
HZ: herpes zoster
LISA: local indicators of spatial association
VZV: varicella-zoster virus
WHO: World Health Organization

## Data Availability

The data sets generated and/or analyzed during the present study are available from the corresponding author on reasonable request.
