# Supplementary figures and images for: Epidemiological Characteristics of Varicella in Anhui Province, China, 2012-2021: Surveillance Study
Source: JMIR Public Health Surveill. 2024 Apr 5;10:e50673. doi: 10.2196/50673 (PMC11031691; doi:10.2196/50673)

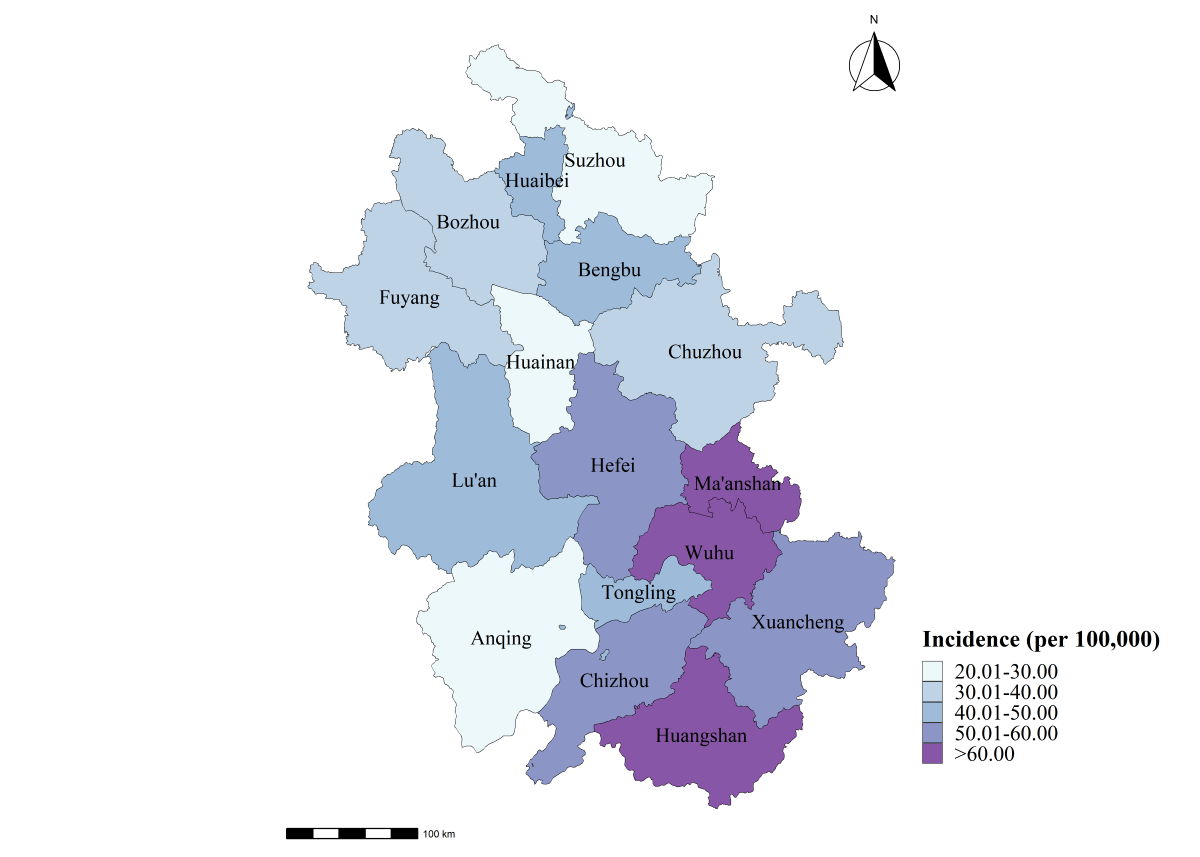

Supplement: Multimedia Appendix 1 [file publichealth_v10i1e50673_app1.png]
